# Validation of elevated levels of interleukin-8 in the cerebrospinal fluid, and discovery of new biomarkers in patients with GBS and CIDP using a proximity extension assay

**DOI:** 10.3389/fimmu.2023.1241199

**Published:** 2023-11-23

**Authors:** Ivan Kmezic, Rasmus Gustafsson, Katharina Fink, Anders Svenningsson, Kristin Samuelsson, Caroline Ingre, Tomas Olsson, Magnus Hansson, Ingrid Kockum, Milena Z. Adzemovic, Rayomand Press

**Affiliations:** ^1^ Department of Neurology, Karolinska University Hospital, Stockholm, Sweden; ^2^ Department of Clinical Neuroscience, Karolinska Institutet, Stockholm, Sweden; ^3^ Department of Clinical Sciences, Karolinska Institutet Danderyd Hospital, Stockholm, Sweden; ^4^ Department of Clinical Chemistry, Karolinska University Hospital, Stockholm, Sweden; ^5^ Department of Laboratory Medicine H5, Karolinska Institutet, Stockholm, Sweden; ^6^ Centre for Neurology, Academic Specialist Centre, Stockholm Health Services, Stockholm, Sweden

**Keywords:** Guillain-Barré syndrome, chronic inflammatory demyelinating polyneuropathy, paraproteinemia-related demyelinating polyneuropathy, multifocal motor neuropathy, interleukin-8, cerebrospinal fluid, plasma, Olink proteomics

## Abstract

**Background:**

Biomarkers for diagnosis of inflammatory neuropathies, assessment of prognosis, and treatment response are lacking.

**Methods:**

CSF and EDTA plasma from patients with Guillain-Barré syndrome (GBS), chronic inflammatory demyelinating polyneuropathy (CIDP), healthy controls (HC) and disease controls were analyzed with Olink multiplex proximity extension assay (PEA) from two independent cohorts. Levels of interleukin-8 (IL8) were further analyzed with ELISA in patients with GBS, CIDP, paraproteinemia-related demyelinating polyneuropathy (PDN), multifocal motor neuropathy (MMN), HC and disease controls. ROC analysis was performed. Outcome was measured with the GBS-disability score (GBS-ds) or Inflammatory Neuropathy Cause and Treatment (INCAT) score.

**Results:**

In CSF, multiplex PEA analysis revealed up-regulation of IL8 in GBS compared to CIDP and HC respectively, and CIDP compared to HC. In addition, levels of IL2RA were upregulated in GBS compared to both HC and CIDP, SELE in GBS compared to HC, and ITGAM, IL6, and NRP1 in GBS compared to CIDP. In plasma, levels of MMP3, THBD and ITGAM were upregulated in CIDP compared to HC. Validation of multiplex IL8 results using ELISA, revealed increased levels of IL8 in CSF in patients with GBS and CIDP versus HC and non-inflammatory polyneuropathies (NIP) respectively, as well as in PDN versus NIP and HC. Levels of IL8 in CSF correlated with impairment in the acute phase of GBS as well as outcome at 6-months follow up.

**Conclusion:**

IL8 in CSF is validated as a diagnostic biomarker in GBS and CIDP, and a prognostic biomarker in GBS. Multiplex PEA hereby identifies several potential biomarkers in GBS and CIDP.

## Introduction

The search for diagnostic and prognostic biomarkers in inflammatory neuropathies is a growing research field (1–3), prompted by the fact that the diagnosis of Guillain-Barré syndrome (GBS) and chronic inflammatory demyelinating polyneuropathy (CIDP) are merely based on clinical features and nerve conduction studies, and lack disease-specific blood and cerebrospinal fluid (CSF) findings (4–6).

Interleukin-8 (IL8) in CSF has recently been proposed as a biomarker to differentiate GBS from CIDP, including acute-onset CIDP (7). Increased levels of IL8 in CSF have also been reported in patients with IgG4 anti-neurofascin 155 antibody positive nodopathy compared to typical CIDP (8). However, comparisons between patients with inflammatory neuropathies and healthy individuals, non-inflammatory neuropathies and other neuroinflammatory disorders has not previously been reported, nor its prognostic value.

IL8 is a proinflammatory chemotactic cytokine of the CXC family and is primarily produced by blood monocytes and tissue macrophages (9, 10), but also by T cells, NK cells, neutrophils, endothelial cells, fibroblasts and various epithelial cells, in response to inflammation and other proinflammatory cytokines (*e.g.*, TNFα, interleukin 1, interleukin 6, CXCL12) (11–13). The main function of IL8 is the recruitment of neutrophils, but also T cells, and dendritic cells to the site of inflammation (11, 13, 14).

The aim of this retrospective study was to investigate the diagnostic and prognostic value of IL8 and related proteins in patients with acute and chronic inflammatory demyelinating neuropathies.

## Materials and methods

Initially, we performed a broad pilot screening with several multiplex proteomic panels (Olink Bioscience, Uppsala) using proximity extension assays (PEA) technology. This technique uses multi-epitope binding for detection which gives a high specificity, while amplification allows for relatively high sensitivity to detect low protein concentrations (15). To further validate our preliminary multiplex proteomics results regarding IL8 and a number of IL8-related proteins, we performed a replication analysis with multiplex proteomics, as well as a routine available enzyme-linked immunosorbent assay (ELISA) test of IL8 in a larger group of patients with inflammatory neuropathies and controls.

### Study cohort

The study was conducted in two phases using three different cohorts (**Table 1**). In the first phase, we used a cohort (Multiplex discovery cohort) consisting of 6 patients with GBS, 6 amyotrophic lateral sclerosis (ALS) and 20 age-matched healthy controls (HC). In the second phase, we used two additional cohorts: the Multiplex replication and ELISA cohorts. The Multiplex replication cohort consisted of 6 patient with GBS, 6 CIDP, 4 non-inflammatory polyneuropathies (NIP), and 6 age-matched HC. The ELISA cohort consisted of 28 patients with GBS, 23 CIDP, 6 paraproteinemia-related demyelinating polyneuropathy (PDN), 6 multifocal motor neuropathy (MMN), and controls. The control groups in ELISA cohort consisted of 44 HC, 18 NIP, 33 pre-treatment multiple sclerosis (MS) sampled at diagnosis prior to treatment, and 31 ALS also sampled at diagnosis. There was no overlap between patients in the multiplex discovery cohort and the other two cohorts, *i.e.*, different samples were analyzed. The NIP group in the Multiplex replication cohort consisted of hereditary demyelinating and axonal neuropathies, and in the ELISA cohort of chronic idiopathic axonal polyneuropathies and hereditary demyelinating and axonal neuropathies.

**Table 1:** Demographics and laboratory findings.

	Multiplex discovery cohort			Multiplex replication cohort				
	GBS	HC	ALS	GBS	CIDP	HC	NIP	
N (total)	6	20	6	6	6	6	4	
Age at sampling*	51.5 ± 12.5	48.8 ± 13.3	53.5 ± 12.8	52.8 ± 16.0	58.3 ± 5.6	51.8 ± 16.9	54.0 ± 10.1	
Sex (m:f)	4:2	12:8	4:3	3:3	4:2	3:3	2:2	
CSF cells (x10^6^/L)*	1.6 ± 0.9	NA	1.3 ± 2.4	1.2 ± 0.9	2.3 ± 2.0	1.3 ± 1 1.0	2.2 ± 0.1	
Q.alb*	15.6 ± 5.0	NA	6.4 ± 1.8	19.6 ± 9.4	11.3 ± 4.7	4.8 ±1.7	5.9 ± 1.8	
OCB, N (%)	0 (0)	NA	0 (0)	0 (0)	0 (0)	0 (0)	0 (0)	
IgG index*	0.58 ± 0.07	NA	0.47 ± 0.03	0.65 ± 0.08	0.57 ± 0.07	0.48 ± 0.06	0.45 ± 0.04	
Infection prior to onset of symptom, N (%)	3 (50)	NA	NA	2 (33.3)	NA	NA	NA	
Comorbidity, N (%) Autoimmune Cancer	0 (0) 0 (0)	0 (0) 0 (0)	0 (0) 0 (0)	0 (0) 0 (0)	0 (0) 0 (0)	0 (0) 0 (0)	0 (0) 0 (0)	
ELISA cohort								
	GBS	CIDP	PDN	MMN	HC	NIP	MS	ALS
N (total)	28	23	6	6	44	18	33	31
N pre-treatment	21	16	6	6	NA	NA	33	NA
N post-treatment	7	7	0	0	NA	NA	0	NA
Age at sampling*	53.9 ± 15.3	58.8 ± 12.6	67.5 ± 10.9	50.6 ± 14.2	43.1 ± 10.3	57.5 ± 15.7	37.5 ± 9.7	62.6 ± 11.3
Sex (m:f)	16 : 12	15 : 8	6 :0	4 : 2	23 : 21	11 : 7	10 : 23	15 : 16
CSF cells (x10^6^/L)*	3.0 ± 2.9	2.2 ± 1.6	2.2 ± 0.9	1.3 ± 0.5	1.9 ± 1.3	2.1 ± 1.3	9.1 ± 15.3	1.8 ± 1.7
Q.alb*	19.1 ± 14.3	12.9 ± 9.1	13.0 ± 4.4	5.4 ± 2.1	4.1 ± 1.5	7.2 ± 2.6	5.2 ± 2.5	6.1 ± 2.6
OCB, N (%)*	0 (0)	1 (4.4)	1 (16.7)	0 (0)	0 (0)	1 (5.6)	33 (100)	1 (3.2)
IgG index *	0.60 ± 0.2	0.52 ± 0.08	0.54 ± 0.05	0.48 ± 0.04	0.48 ± 0.04	0.49 ± 0.09	1.1 ± 0.4	0.47 ± 0.05
Paraprotein, N (%)	0 (0)	8 (34.8)^1^	6 (100)^2^	0 (0)	NA	NA	NA	NA
Ganglioside abs, N (%)	2 (7.1)^3^	2 (8.7)^4^	1 (16.7)^4^	3 (50)^5^	NA	NA	NA	NA
Infection prior to onset of symptom, N (%)	13 (46.4)	NA	NA	NA	NA	NA	NA	NA
Comorbidity, N (%) Autoimmune Cancer	0 (0) 1 (3.6)^6^	1 (4.4)^7^ 1 (4.4)^6^	0 (0) 1 (16.7)^6^	0 (0) 0 (0)	0 (0) 0 (0)	1 (5.6)^7^ 0 (0)	0 (0) 0 (0)	1 (3.2)^7^ 1 (3.2)^6^

GBS, Guillain-Barré syndrome; CIDP, chronic inflammatory demyelinating polyneuropathy; PDN, paraproteinemia-related demyelinating polyneuropathy; MMN, multifocal motor neuropathy; HC, healthy controls; NIP, non-inflammatory polyneuropathies (the NIP group in the Multiplex replication cohort consisted of hereditary demyelinating and axonal neuropathies, and in the ELISA cohort of chronic idiopathic axonal polyneuropathies and hereditary demyelinating and axonal neuropathies); MS, multiple sclerosis; ALS, amyotrophic lateral sclerosis; Q.alb, albumin quotient (CSF/plasma quotient of albumin); OCB, oligoclonal bands; NA, not applicable; abs, antibodies.

^1^IgG or IgA, ^2^IgM, of which all with MAG-abs, ^3^ IgG anti-GQ1b, ^4^IgM anti-GD1b, IgM anti-GM1, IgM anti-GM2, IgM anti-GQ1b, ^5^IgM anti-GM1, ^6^melanoma 22 years prior to GBS, prostate cancer 5 years prior to CIDP, and 3 years prior to PDN, breast cancer 6 years prior to ALS, ^7^Graves’ disease 6 years prior to CIDP, 6 years prior to ALS, 5 years prior to sample collection in patient with NIP; *mean ± standard deviation (SD).

In the ELISA cohort, 7 patients with GBS and 7 patients with CIDP underwent a second lumbar punction (LP) after initiation of immunomodulatory treatment. These samples were classified as post-treatment.

The diagnosis of GBS was based on Asbury criteria (4) and/or Brighton criteria (level 1 or level 2 of diagnostic certainty) (5), whereas the CIDP, PDN and MMN diagnoses were based on the EFNS criteria from 2010 (16–18). The diagnosis of MS and ALS was based on well-recognized international criteria (19, 20).

Demographics, clinical features, electrodiagnostic and laboratory findings of all cohorts are summarized in **Tables 1**, **2**.

**Table 2:** Clinical features and electrodiagnostic findings.

	GBS			CIDP		PDN	MMN
	Multiplex discovery cohort	Multiplex replication cohort	ELISA cohort	Multiplex replication cohort	ELISA cohort	ELISA confirmation cohort	ELISA cohort
Electrodiagnostic tests	4 AIDP 2 equivocal	3 AIDP 2 AMSAN 1 equivocal	10 AIDP 2 AMAN 3 AMSAN 5 equivocal 8 others *	demyelination	demyelination	demyelination	demyelination
Phenotype	6 typical	6 typical	20 typical 8 others *	4 typical 1 distal 1 motor-dominant	16 typical 3 MADSAM 1 distal 1 motor-dominant 2 acute-onset	typical	typical

GBS, Guillain-Barré syndrome; CIDP, chronic inflammatory demyelinating polyneuropathy; PDN, paraproteinemia-related demyelinating polyneuropathy, MMN, multifocal motor neuropathy; AIDP, acute inflammatory demyelinating polyneuropathy; AMSAN, acute motor sensory axonal neuropathy; AMAN, acute motor axonal neuropathy; MADSAM, multifocal acquired demyelinating sensory and motor neuropathy (Lewis–Sumner syndrome); ELISA, enzyme-linked immunosorbent assay.

*2 patients with Miller-Fisher syndrome (MFS), 2 overlap MFS/GBS, 1 overlap pharyngeal–cervical–brachial variant/GBS; 3 subacute inflammatory demyelinating polyneuropathy (SIDP).

### Patient samples

All patients and controls (except HC) were diagnosed at the Department of Neurology at Karolinska University Hospital, Stockholm. The time of sampling in all three cohorts was same as the time of diagnosis, i.e., the samples were collected during the diagnostic workup. The only exception were samples collected post-treatment in the ELISA cohort in some patients with GSB and CIDP as presented in **Table 1**. CSF and EDTA plasma samples from patients and controls were handled in a similar manner and were stored at the biobank of Department of Neurology between 2005 and 2017. Samples from HC used in Multiplex and ELISA cohort were collected at the Department of Neurology, University Hospital of Umeå between 2012 and 2013. CSF and plasma were centrifuged at 2000 g for 10 min and 400 μl of each was aliquoted into glass tubes and stored at −80°C. All samples were thawed once and aliquoted before shipment to the Olink laboratory, Uppsala, and the laboratory of the Department of Clinical Chemistry, Karolinska University Hospital.

### Multiplex proteomics

The CSF and plasma proteins were measured using all available (thirteen) Olink panels in 2018. Each assay panel consisted of ninety-two protein targets (92 + 4 controls), resulting in a total of >1100 protein measures per sample and per patient. Protein quantification was performed using PEA technology, which utilizes paired oligonucleotide antibody probes and quantitative polymerase chain reaction (qPCR) for detection and quantification (15, 21). A mixture of antibody probes was incubated with each sample, i.e., two matched antibodies, labeled with DNA oligonucleotides bound to a target protein in solution, This brought the two antibodies into proximity, which allowed DNA oligonucleotides to hybridize and extend by a DNA polymerase. After hybridization and extension this newly created piece of DNA, which is unique for the specific antigen, was amplified by PCR. Following binding, hybridization, extension, and amplification protein levels were quantified using qPCR and expressed on a relative logarithmic scale with two as a base (log2) and with arbitrary units presented as normalized protein expression (NPX). Proteins with call rate <60%, i.e., proteins detected in less than 60% of the samples were excluded from further analyses. The call rates for proteins that passed quality control, their main function and alternative names (synonyms), as well as the list of all panels that were analyzed are provided in **Supplementary Table 1** in the **Supplementary Material**.

### ELISA

Levels of IL8 in CSF and plasma were analyzed at the laboratory of the Department of Clinical Chemistry, Karolinska University Hospital using a Chemiluminescence assay on an Immulite 1000 instrument from Siemens (Siemens Healthcare Diagnostics Products GmbH, Marburg, Germany). Measuring range 2-7500 ng/L. Imprecision expressed as CV 7,2% at 109 ng/L, and 6,4% at 484 ng/L.

### Clinical data and assessment

Clinical data were retrieved from the medical chart e-health system TakeCare and the national Swedish Neuro Registries (https://www.neuroreg.se). Patients with GBS were assessed with the GBS-disability scale (GBS-ds) (22) on the day of CSF/blood sampling, at nadir, 3 months ( ± 1 week), 6 months ( ± 2 weeks), and 12 months ( ± 4 weeks) later. The Erasmus GBS outcome score (EGOS) (23) was calculated for patients with GBS. Patients with CIDP, PDN, and MMN were assessed with the Inflammatory Neuropathy Cause and Treatment (INCAT) (24) scale on the day of CSF/blood sampling, at the above mentioned time points, and additionally at 18 months, ( ± 4 weeks), 24 months ( ± 4 weeks), 5 years ( ± 2 months), and 8–10 years post-onset.This study was approved by Regional Ethical Review Board of Stockholm (EPN 2014/1815-31/4 and 2017/952–31/1) and Umeå Ethical Review Board (EPN 2011–39-31 M).

### Statistics

The multiplex proteomics data and associations between protein levels were determined using a multi-variable linear regression model adjusted for sex and age at sampling, as previously described (25). Protein measures were analyzed with the default log base-two transformed protein levels and illustrated through volcano plots, negative 10log based transformed significance over NPX-based beta-coefficient. Associations were additionally corrected for multiple testing using a false discovery rate (FDR) approach using a FDR-corrected significance of p_FDR_<0.05. Considering sample handling issues, the model was also corrected with markers used for assessing sample handling (CCL19 for CSF and CD40L for plasma) (25, 26).

The normality of ELISA IL8 data distribution was assessed with the Anderson-Darling test, D`Agostino & Pearson test, Shapiro-Wilk test, and Kolmogorov-Smirnov test. If the distribution was non-Gaussian, a log-based transformation was used before performing multiple comparisons. If the distribution was non-Gaussian or if the sample size was small, multiple comparisons were performed with the Kruskal-Wallis test. Otherwise, multiple comparisons were performed using one-way ANOVA. Multiple comparisons were corrected with Holm-Sidak or Dunn’s test. Likewise, depending on data distribution, correlation analysis was done by calculating Pearson or Spearman correlation coefficient. To further evaluate the diagnostic value of IL8 receiver operating characteristic (ROC) curve analysis was performed, and the area under the curve (AUC), sensitivity, specificity, and likelihood ratio (LR) were calculated. To evaluate the association between levels of IL8 and clinical outcome, Fisher´s exact test and logistic regression analysis were performed.

All statistical analyses were performed, and figures computed in R-4.1.3 and GraphPad Prism 8.0.

## Results

### IL8 in the multiplex cohorts in patients with GBS and CIDP

**Figure 1:**
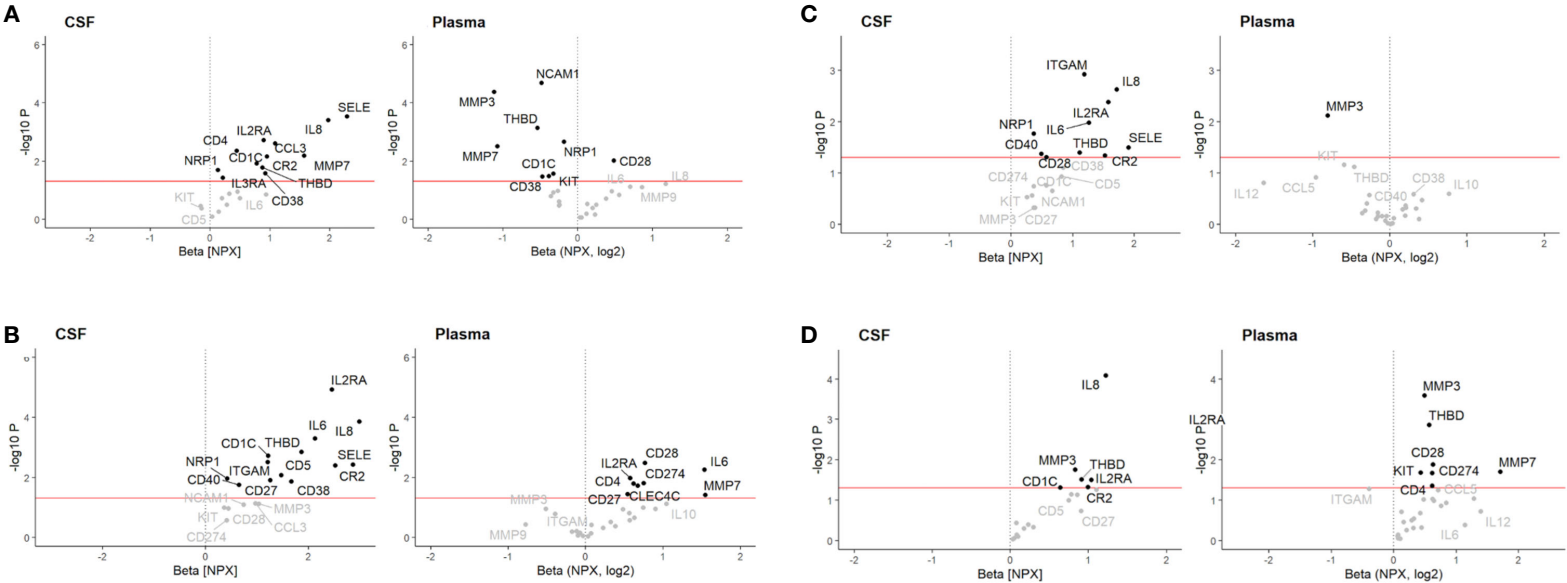
Volcano plot illustrating differential cerebrospinal fluid (CSF) and plasma protein levels associated with **(A)** Guillain-Barré syndrome (GBS) relative to healthy controls (HC) in Multiplex discovery cohort, **(B)** GBS relative to HC in Multiplex replication cohort, **(C)** GBS relative to chronic inflammatory demyelinating polyneuropathy (CIDP), and **(D)** CIDP relative to HC. Associations were assessed using a multivariable linear regression model adjusted for sex and age at sampling. Red line: p < 0.05. NPX, log2 = relative log2 scale presented as normalized protein expression (NPX).

After correcting for multiple comparisons and using the model corrected with the additional sample handling markers, we found that pre-treatment levels of IL8 in CSF, but not plasma, in both Multiplex cohorts were upregulated in patients with GBS compared to all other groups, *i.e*., HC, CIDP, NIP and ALS (p_FDR_ < 0.05) (**Figures 1A–C**; **Table 3**). Furthermore, we also found that pre-treatment levels of IL8 in CSF, but not plasma, were upregulated in patients with CIDP compared to HC (p_FDR_ < 0.05) (**Figure 1D**; **Table 3**).

**Table 3:** Interleukin-8 (IL8) levels associated with GBS and CIDP.

	Model corrected for age and sex				Model corrected for age, sex and sample handling variability			
	Beta	SE	p	p_FDR_	Beta	SE	p	p_FDR_
IL8 in CSF								
GBS versus HC **^1^**	1.97	0.426	0.0004	0.0043*	1.66	0.415	0.0015	0.0163*
GBS v. HC **^2^**	2.98	0.438	0.0001	0.0013*	2.62	0.462	0.0008	0.0071*
GBS v. CIDP	1.71	0.39	0.0024	0.0223*	1.71	0.419	0.0046	0.0291*
GBS v. NIP	3.28	0.723	0.0039	0.0362*	2.96	0.837	0.0167	0.0444*
GBS v. ALS	1.94	0.512	0.0053	0.0386*	1.61	0.342	0.0022	0.0159*
CIDP v. HC	1.22	0.166	8.08e-05	0.0015*	1.13	0.181	0.0004	0.0081*
CIDP v. NIP	1.7	0.607	0.0313	0.149	2.08	0.93	0.0759	0.406
IL8 in plasma								
GBS v. HC ^1^	1.17	0.585	0.0607	0.17	0.856	0.487	0.0979	0.211
GBS v. HC ^2^	-0.101	0.535	0.854	0.913	-0.214	0.353	0.564	0.711
GBS v. CIDP	-0.0275	0.369	0.942	0.986	0.00252	0.421	0.995	0.995
GBS v. NIP	0.538	0.447	0.274	0.398	0.99	1.05	0.388	0.922
GBS v. ALS	1.09	0.9	0.262	0.612	0.0527	0.779	0.948	0.996
CIDP v. HC	0.0673	0.423	0.878	0.901	-0.305	0.29	0.328	0.528
CIDP v. NIP	0.693	0.325	0.0768	0.168	0.964	0.645	0.195	0.567

Associations were assessed using two different multi-variable linear regression models (to the left corrected for age and sex; to the right corrected for age, sex and sample handling variability).

GBS, Guillain-Barré syndrome; CIDP, chronic inflammatory demyelinating polyneuropathy; HC, healthy controls; NIP, non-inflammatory polyneuropathies; ALS, amyotrophic lateral sclerosis; SE, standard error; p, p-value; p_FDR_, false discovery rate adjusted p-value.

^1^Olink discovery cohort; **^2^** Olink replication cohort; *statistically significant.

### Discovery of novel biomarkers other than IL8 in the Multiplex cohorts in patients with GBS and CIDP

In this study, we focused particulary on those protein markers pertaining to IL8 and its pathway.

#### GBS compared to HC

In CSF, thirteen proteins were upregulated in GBS, of which six (SELE, IL2RA, CR2, CD1C, THBD, NRP1) were successfully validated in both cohorts (p_FDR_ < 0.5) (**Figures 1A, B**; **Table 4A**). However, only SELE and IL2RA remained significantly upregulated in the model adjusted even for sample handling (**Table 4B**). In plasma, the findings were more inconsistent, even if several proteins exhibited association with GBS in the discovery cohort (p_FDR_ < 0.05), but not after the FDR correction in the replication cohort.

**Table 4:** Protein levels associated with GBS compared to HC in the regression model adjusted for sex and age (A), and in the regression model adjusted for sex, age and sample handling (B) in multiplex cohorts.

A								
	Multiplex discovery cohort				Multiplex replication cohort			
	Beta	SE	p	p_FDR_	Beta	SE	p	p_FDR_
Proteins in CSF								
SELE **^#^**	2.28	0.476	0.0003	0.0043*	2.85	0.705	0.0037	0.0095*
IL2RA **^#^**	0.894	0.234	0.0019	0.0134*	2.45	0.256	1.18e-05	0.0002*
CCL3	1.08	0.294	0.0025	0.0134*	0.966	0.47	0.0738	0.095
CD4	0.443	0.131	0.0043	0.0191*	NA	NA	NA	NA
MMP7	1.57	0.49	0.0065	0.0217*	NA	NA	NA	NA
CR2	0.947	0.299	0.0069	0.0217*	2.52	0.631	0.0041	0.0095*
CD1C	0.773	0.268	0.0119	0.0328*	1.21	0.266	0.0019	0.0071*
THBD	0.87	0.319	0.0163	0.0398*	1.86	0.39	0.0014	0.0067*
NRP1	0.125	0.0477	0.0198	0.0435*	0.418	0.127	0.0109	0.0206*
CD38	0.918	0.369	0.0259	0.0519	1.66	0.507	0.0136	0.0216*
IL3RA	0.208	0.0898	0.0371	0.0681	NA	NA	NA	NA
IL6	0.931	0.597	0.141	0.207	2.12	0.377	0.0005	0.0032*
ITGAM	NA	NA	NA	NA	1.2	0.288	0.0031	0.0095*
CD5	0.035	0.14	0.807	0.807	1.46	0.42	0.0083	0.0175*
Proteins in plasma								
MMP7	-1.08	0.315	0.0031	0.0172*	1.54	0.625	0.0389	0.141
CD28	0.48	0.166	0.0096	0.0449*	0.761	0.174	0.0033	0.0808
NCAM1	-0.487	0.0853	2.03e-05	0.0006*	-0.0731	0.191	0.713	0.823
MMP3	-1.12	0.208	4.13e-05	0.0006*	-0.511	0.286	0.112	0.254
THBD	-0.54	0.133	0.0007	0.0067*	0.0702	0.203	0.738	0.823
NRP1	-0.188	0.0524	0.0022	0.015*	0.0323	0.286	0.913	0.913
B								
	Multiplex discovery cohort				Multiplex replication cohort			
	Beta	SE	p	p_FDR_	Beta	SE	p	p_FDR_
Proteins in CSF								
SELE	1.79	0.377	0.0004	0.0086*	2.33	0.766	0.0188	0.0446*
IL2RA	0.741	0.234	0.0075	0.0466*	2.47	0.313	9.94e-05	0.0019*
CCL3	0.836	0.27	0.0085	0.0466*	0.684	0.534	0.241	0.294
CD4	0.359	0.131	0.0169	0.0743	NA	NA	NA	NA
MMP7	1.2	0.47	0.0245	0.0786	NA	NA	NA	NA
CR2	0.692	0.273	0.025	0.0786	1.92	0.624	0.0178	0.0446*
CD1C	0.561	0.252	0.0446	0.123	1.04	0.299	0.0102	0.0388*
THBD	0.656	0.317	0.059	0.144	1.92	0.477	0.0051	0.0238*
NRP1	0.0965	0.0485	0.0683	0.15	0.288	0.119	0.0459	0.0793
CD38	0.635	0.352	0.0943	0.173	1.18	0.447	0.0389	0.0738
Proteins in CSF								
IL3RA	0.183	0.0982	0.0867	0.173	NA	NA	NA	NA
IL6	0.37	0.514	0.485	0.547	1.89	0.427	0.0031	0.0194*
ITGAM	NA	NA	NA	NA	1.15	0.351	0.0136	0.0429*
CD5	-0.0882	0.126	0.497	0.547	1.51	0.514	0.0218	0.046*
Proteins in plasma								
MMP7	-1.09	0.334	0.0047	0.0263*	1.6	0.628	0.0381	0.138
CD28	0.391	0.149	0.0179	0.0717	0.76	0.188	0.0067	0.0487*
NCAM1	-0.53	0.0784	3.32e-06	9.29e-05*	-0.0829	0.202	0.693	0.773
MMP3	-1.18	0.212	3.58e-05	0.0005*	-0.555	0.253	0.0648	0.166
THBD	-0.573	0.136	0.0006	0.0055*	0.0666	0.217	0.768	0.825
NRP1	-0.199	0.0542	0.0019	0.0134*	0.0074	0.29	0.98	0.98

A) Associations were assessed using a multi-variable linear regression model adjusted for sex and age at sampling.

Also significally upregulated in discovery and replication cohort in the model adjusted for sample handling (see **Table 4B**).

B) Associations were assessed using a multi-variable linear regression model adjusted for sex, age and sample handling.

GBS, Guillain-Barré syndrome; HC, healthy controls; SE, standard error; p, p-value; p_FDR_, false discovery rate adjusted p-value; NA = not applicable due to low call rate (<60%).

*statistically significant.

See also **Supplementary Table S1** for protein abbreviation presented in this table.

#### GBS compared to CIDP

In CSF, nine proteins were upregulated in GBS, of which four (ITGAM, IL2RA, IL6, NRP1) were significant also after FDR correction in the model also adjusted for sample handling (p_FDR_ < 0.5). In plasma, down-regulation of two proteins exhibited association with GBS, but they did not remain significant after FDR correction (**Figure 1C**; **Table 5**).

**Table 5:** Protein levels associated with GBS compared to CIDP.

	Model corrected for age and sex				Model corrected for age, sex and sample handling variability			
	Beta	SE	p	p_FDR_	Beta	SE	p	p_FDR_
Proteins in CSF								
ITGAM	1.19	0.242	0.0012	0.0223*	1.19	0.26	0.0026	0.0291*
IL2RA	1.57	0.397	0.0042	0.0262*	1.58	0.427	0.0077	0.0364*
IL6	1.26	0.38	0.0104	0.0495*	1.33	0.315	0.0039	0.0291*
NRP1	0.369	0.123	0.017	0.0647	0.387	0.114	0.0116	0.0441*
SELE	1.9	0.73	0.0315	0.0933	1.91	0.785	0.0455	0.108
THBD	1.11	0.452	0.0398	0.0933	1.09	0.481	0.0577	0.11
CD40	0.487	0.202	0.0421	0.0933	0.519	0.183	0.0254	0.0804
CR2	1.52	0.639	0.045	0.0933	1.56	0.669	0.0527	0.11
CD28	0.57	0.246	0.0491	0.0933	0.557	0.26	0.0693	0.12
Proteins in plasma								
MMP3	-0.809	0.228	0.0076	0.22	-0.688	0.229	0.0197	0.449
KIT	-0.593	0.276	0.0688	0.732	-0.993	0.354	0.031	0.449

Associations were assessed using two different multi-variable linear regression models (corrected for age and sex and corrected for age, sex and sample handling variability).

GBS, Guillain-Barré syndrome; CIDP, chronic inflammatory demyelinating polyneuropathy; HC, healthy controls; SE, standard error; p, p-value; p_FDR_, false discovery rate adjusted p-value.

*statistically significant.

See also **Supplementary Table S1** for protein abbreviation presented in this table.

#### CIDP compared to HC

In CSF, five proteins were upregulated in CIDP, but none remained significant after FDR correction. In plasma, eight proteins were associated with CIDP, of which three (MMP3, THBD, ITGAM) were significant also after FDR correction in the model corrected for age, sex and sample handling (**Figure 1D**; **Table 6**).

**Table 6:** Protein levels associated with CIDP compared to HC.

	Model corrected for age and sex				Model corrected for age, sex and sample handling variability			
	Beta	SE	p	p_FDR_	Beta	SE	p	p_FDR_
Proteins in CSF								
MMP3	0.83	0.277	0.0173	0.148	0.57	0.238	0.0477	0.181
THBD	0.913	0.348	0.0304	0.148	1.04	0.397	0.0341	0.181
IL2RA	1.04	0.397	0.0312	0.148	1.16	0.458	0.0387	0.181
CR2	0.99	0.422	0.0468	0.149	0.893	0.493	0.113	0.315
CD1C	0.636	0.273	0.0483	0.149	0.765	0.303	0.0396	0.181
Proteins in plasma								
MMP3	0.493	0.0795	0.0003	0.0075*	0.445	0.0754	0.0006	0.0087*
THBD	0.566	0.118	0.0014	0.0197*	0.581	0.134	0.0035	0.0334*
CD28	0.629	0.198	0.013	0.102	0.601	0.225	0.0319	0.185
MMP7	1.7	0.586	0.0197	0.102	2.01	0.592	0.0116	0.0838
KIT	0.429	0.144	0.0208	0.102	0.341	0.133	0.0433	0.198
CD274	0.612	0.214	0.021	0.102	0.474	0.198	0.0478	0.198
CD4	0.614	0.257	0.0441	0.18	0.478	0.258	0.107	0.344
ITGAM	-0.4	0.175	0.0522	0.18	-0.577	0.0775	0.0001	0.0042*

Associations were assessed using two different multi-variable linear regression models (corrected for age and sex and corrected for age, sex and sample handling variability).

GBS, Guillain-Barré syndrome; CIDP, chronic inflammatory demyelinating polyneuropathy; HC, healthy controls; SE, standard error; p, p-value; p_FDR_, false discovery rate adjusted p-value.

*statistically significant.

See also **Supplementary Table S1** for protein abbreviation presented in this table.

### IL8 in the ELISA cohort in patients with inflammatory neuropathies

Considering results from both the discovery and replication multiplex cohorts showing increased levels of IL8 in CSF, but not plasma, we then analyzed CSF from a larger group of patients with acute and chronic inflammatory neuropathies, and controls using the ELISA test. With regards to plasma, we examined only five patients with GBS and five HC and found the same result as in the Multiplex cohorts, *i.e.*, no statistical difference between these two groups (p > 0.05).

**Figure 2:**
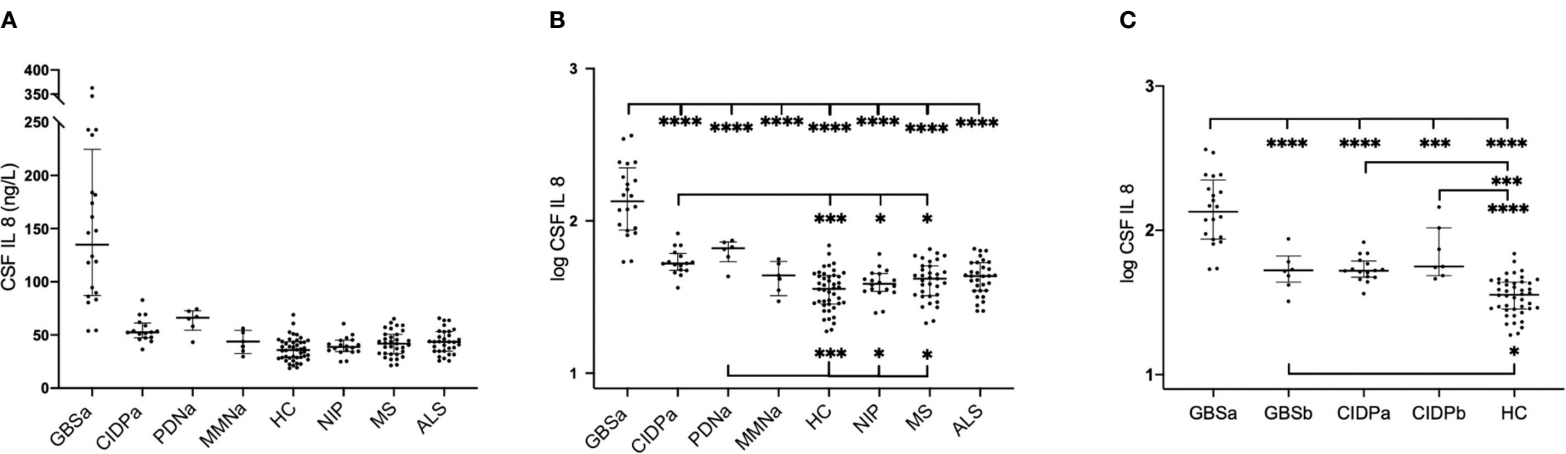
Interleukin-8 (IL8) in cerebrospinal fluid (CSF) in patients with inflammatory neuropathies and controls in the ELISA cohort. Multiple comparisons were assessed using one-way ANOVA. **(A)** Absolute concentrations of pre-treatment levels of IL8; horizontal lines represent median values with interquartile range, **(B)** Logarithmic transformation of pre-treatment values of IL8, **(C)** Logarithmic transformed values of IL8 in GBS and CIDP prior to and following immunomodulatory therapy. *p < 0.05, **p < 0.01, ***p < 0.001, ****p < 0.0001. GBSa, pre-treatment Guillain-Barré syndrome; GBSb, post-treatment Guillain-Barré syndrome; CIDPa, pre-treatment chronic inflammatory demyelinating polyneuropathy; CIDPb, post-treatment chronic inflammatory demyelinating polyneuropathy; PDNa, pre-treatment paraproteinemia-related demyelinating polyneuropathy; MMNa, pre-treatment multifocal motor neuropathy; HC, healthy controls; NIP, non-inflammatory polyneuropathies; MS, multiple sclerosis; ALS, amyotrophic lateral sclerosis; p, p value. *p < 0.05, **p < 0.01, ***p < 0.001, ****p < 0.0001.

In patients with GBS, pre-treatment median levels of IL8 in CSF were significantly higher compared to all groups of patients with chronic inflammatory neuropathies, HC, and all other control groups (**Figure 2**). Post-treatment levels were lower than pre-treatment levels (p < 0.0001), but higher compared to HC (p<0.03).

Comparing the levels of IL8 in patients with subacute inflammatory demyelinating polyneuropathy (SIDP; only 3 cases) to those with CIDP, we found that patients with SIDP had higher levels compared to patients with CIDP (Mann-Whitney p = 0.0008).

In patients with CIDP, pre-treatment median levels of IL8 in CSF were significantly higher compared to HC, NIP, and MS, but lower compared to GBS. Post-treatment levels were as high as pre-treatment levels and significantly higher than HC (p < 0.0001).

In patients with PDN, pre-treatment median levels of IL8 in CSF showed a similar pattern to CIDP. In patients with MMN, pre-treatment levels of IL8 in CSF did neither differ from HC, nor other disease controls (**Figure 2**).

### Time between symptom onset and sampling, and correlation with levels of IL8

In patients with GBS, the time period between symptom onset and sampling was 9 days (median, IQR 4-14; mean 9.3, SD 5.1). In patients with CIDP, the time period between symptom onset and sampling was 9 months (median, IQR 6-12; mean 8.4, SD 3.6).

We did not find any correlation between time of symptom onset and levels of IL8 in CSF (GBS Spearman coefficient r = 0.18, p = 0.43; CIDP Spearman coefficient r = 0.23, p = 0.39).

### ROC analysis in the ELISA cohort

**Figure 3:**
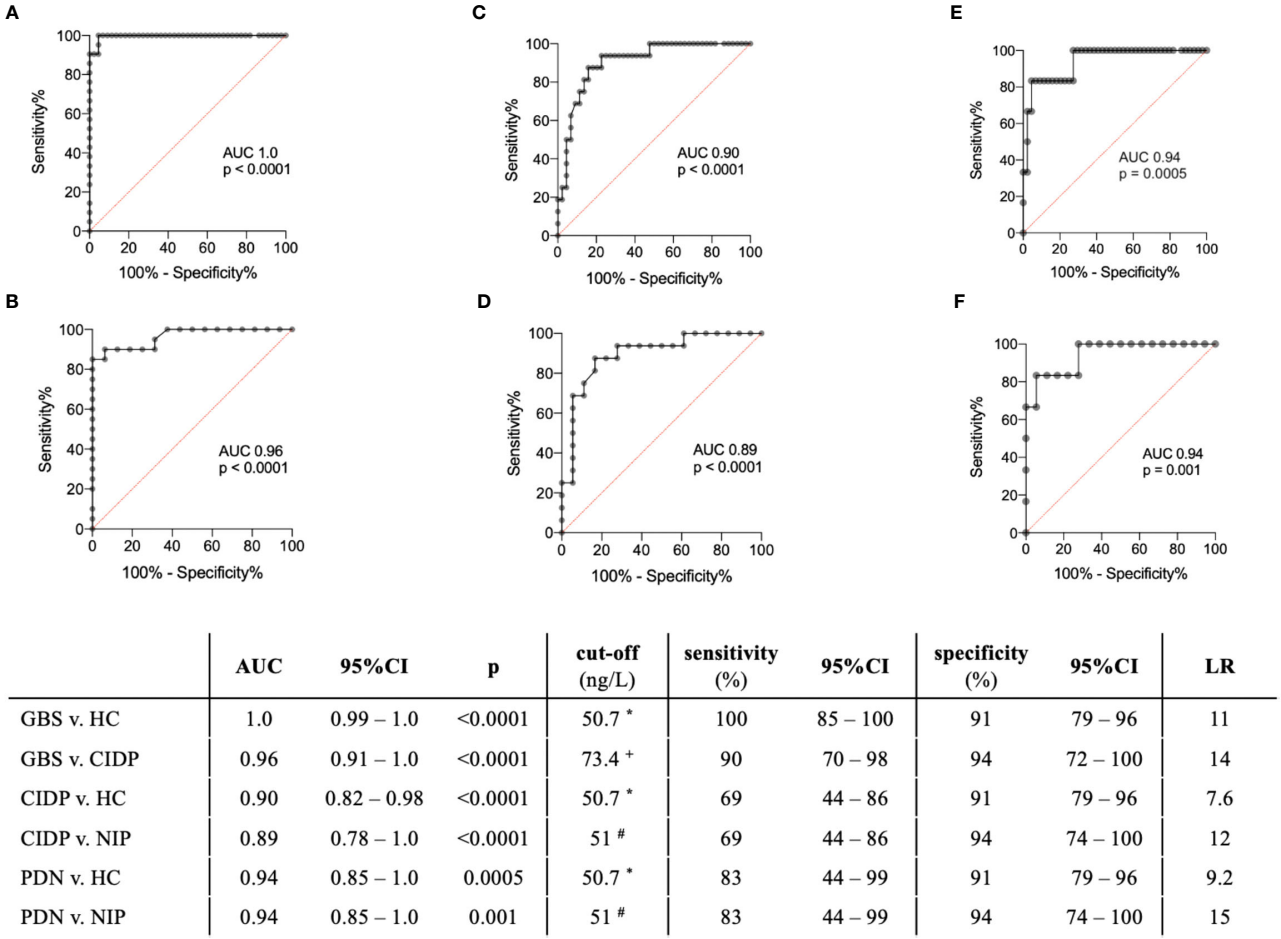
Receiver operating characteristic (ROC) analysis in patients with GBS, CIDP and PDN in the ELISA cohort. **(A)** GBS versus HC, **(B)** GBS v. CIDP, **(C)** CIDP v. HC, **(D)** CIDP v. NIP, **(E)** PDN v. HC, **(F)** PDN v. NIP. * 90% percentile of the HC group (50.7 ng/L). + 90% percentile of the CIDP group (73.4 ng/L). # 90% percentile of the NIP group (51 ng/L). GBS, pre-treatment Guillain-Barré syndrome; CIDP, pre-treatment chronic inflammatory demyelinating polyneuropathy; PDN, pre-treatment paraproteinemia-related demyelinating polyneuropathy; HC, healthy controls; NIP, non-inflammatory polyneuropathies; AUC, area under the curve; p, p value.; LR, likelihood ratios.

ROC analysis comparing levels of IL8 in CSF in GBS versus HC, GBS vs. CIDP, CIDP vs. HC, CIDP vs. NIP, PDN vs. HC, and PDN vs. NIP, yielded AUC values of 1.0, 0.96, 0.90, 0.89, 0.94, and 0.94, respectively. Ninety-five percent confidential interval, p-values, cut-offs, sensitivity, specificity, and likelihood ratios (LR) are summarized in **Figure 3**. An IL8 concentration of 73 ng/L in CSF was identified as cut-off to differentiate patients with GBS from those with CIDP, and 51 ng/L to differentiate CIDP and PDN from non-inflammatory polyneuropathies (**Figure 3**).

### IL8 and disease severity in patients with GBS in the ELISA cohort

**Figure 4:**
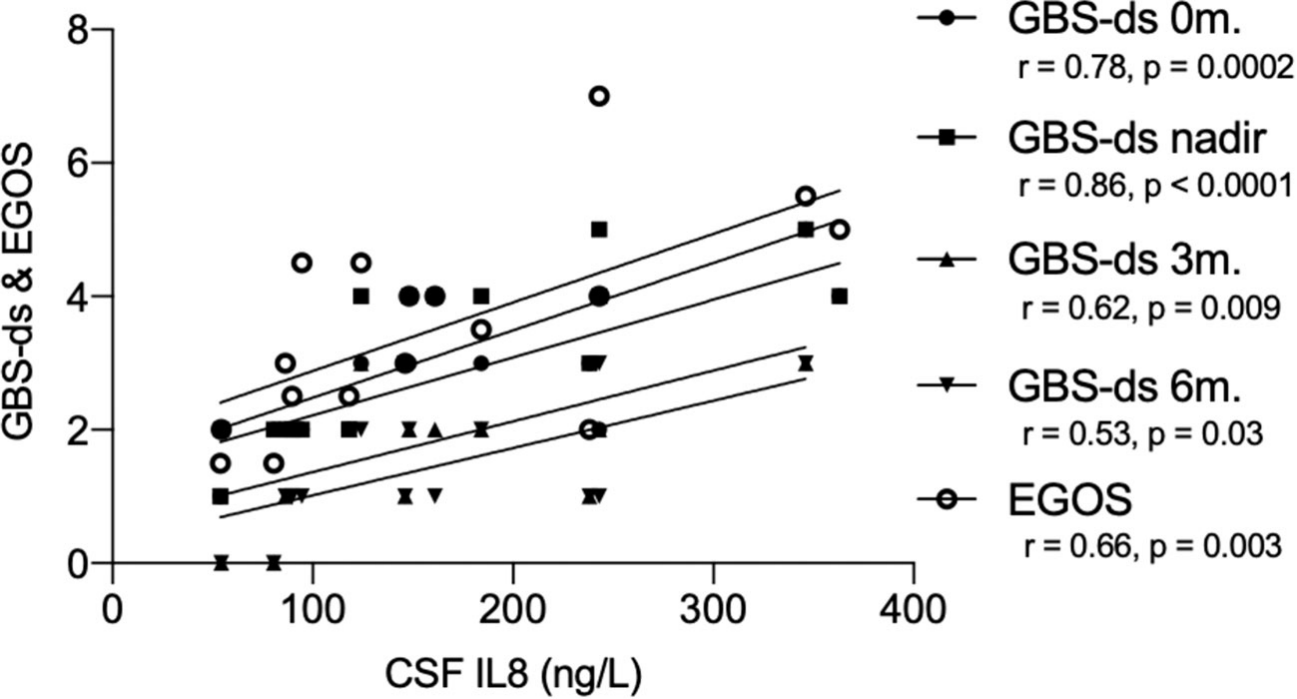
Correlation between pre-treatment levels of interleukin-8 (IL8), and GBS-disability score and the Erasmus GBS outcome score in the ELISA cohort. GBS-ds, Guillain-Barré syndrome disability score; EGOS, the Erasmus GBS outcome score; r, Spearman correlation coefficient; CSF, cerebrospinal fluid; IL8, interleukin-8; 0m., sampling time; 3m., 3-months follow up; 6m., 6-months follow up.

**Figure 5:**
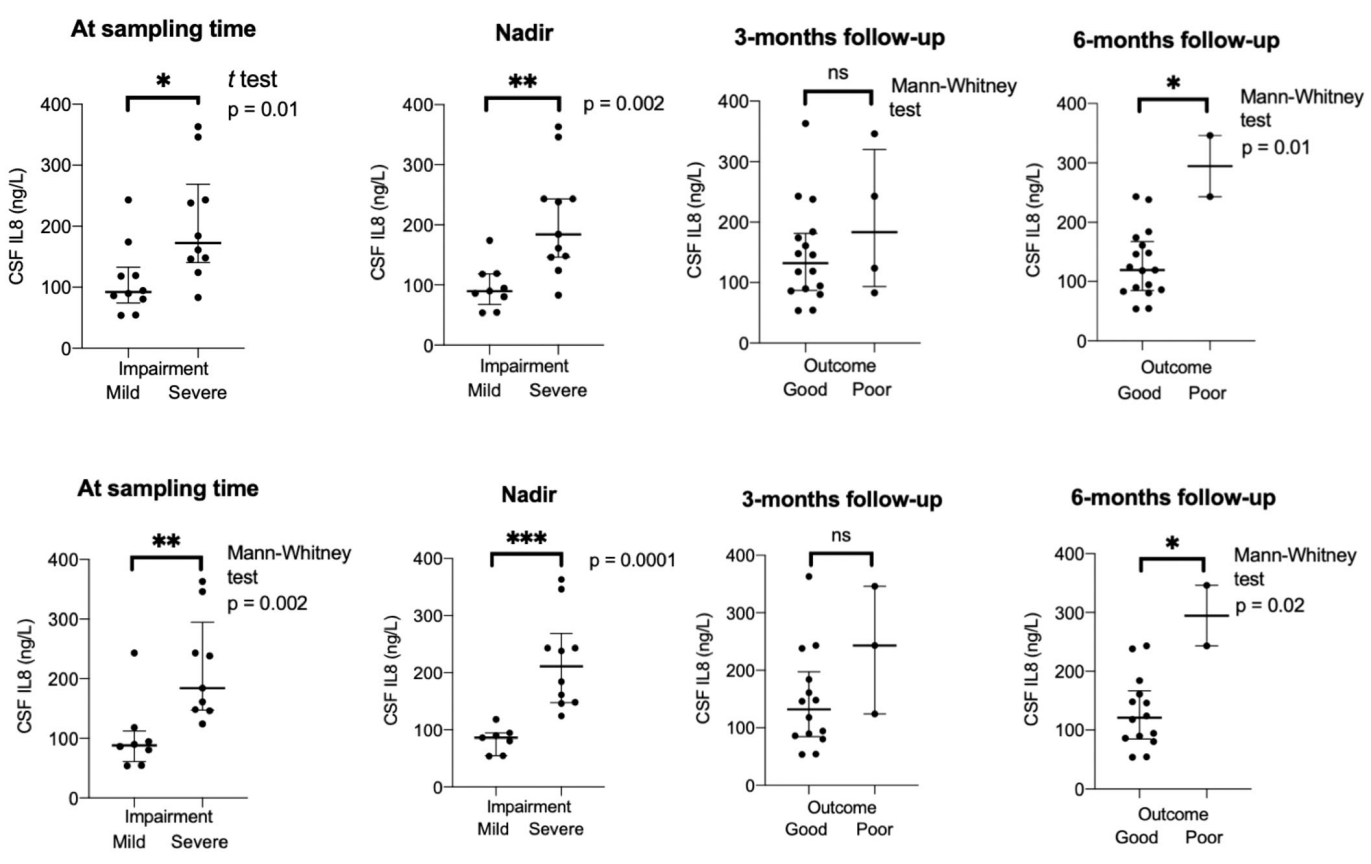
Group comparison mild versus severe impairment in acute phase, and good versus poor outcome at different timepoints in patients with Guillain-Barré syndrome in the ELISA cohort. Bottom row when Miller-Fisher syndrome excluded. CSF, cerebrospinal fluid; IL8, interleukin-8; 0m., sampling time; severe impairment = GBS-ds 3 points or higher (inability to walk unaided); poor outcome = GBS-ds 3 points or higher (inability to walk unaided). *p < 0.05, **p < 0.01, ***p < 0.001. ns, not significant.

We found a correlation between pre-treatment levels of IL8, and disease severity measured with GBS-ds at the time of sampling (Spearman coefficient r = 0.78, p = 0.0002), and nadir (r = 0.86, p < 0.0001), as well as outcome at 3-months follow-up (r = 0.62, p = 0.009), 6-months (r = 0.53, p = 0.03), and EGOS prognostic score (r = 0.66, p = 0.003) (**Figure 4**). Taking GBS-ds of 3 points (inability to walk unaided) as a cut-off for severe impairment in the acute phase of the disease, we found that the group with a more severe impairment had significantly higher pre-treatment levels of IL8 compared to the group with a milder impairment at the time of sampling (p = 0.01), and at nadir (p = 0.002). The same result was found after excluding patients with MFS (p = 0.002, p = 0.0001 respectively) (**Figure 5**).

### Association between pre-treatment levels of IL8 and impairment at nadir in GBS

**Figure 6:**
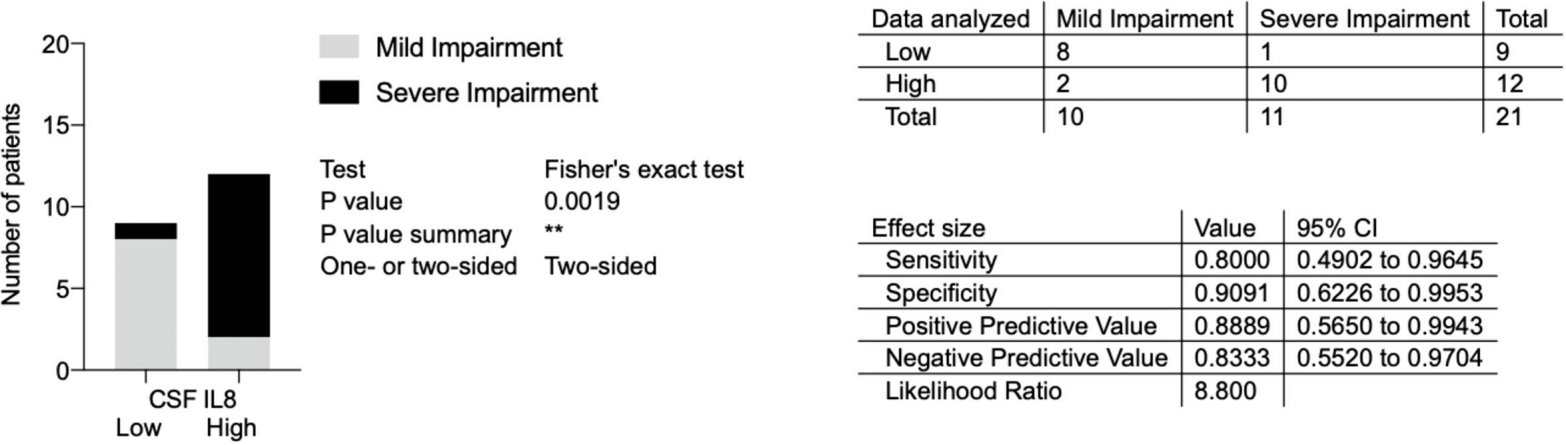
Association between pre-treatment levels of interleukin-8 (IL8) and impairment at nadir in patients with Guillain-Barré syndrome in the ELISA cohort. Low versus high pre-treatment levels of IL8 (cut-off based on GBS group median, 135 ng/L); mild versus severe impairment at nadir (severe impairment = GBS-ds 3 points or higher, *i.e.*, inability to walk unaided). IL8, interleukin-8; CSF, cerebrospinal fluid; GBS, Guillain-Barré syndrome; GBS-ds, GBS-disability scale. **p < 0.01.

To investigate if the higher pre-treatment levels of IL8 is a factor predictive of more severe impairment at the nadir, a Fisher´s test and logistic regression analysis were performed. The GBS group median (135 ng/L) was defined as the cut-off between low versus high levels of IL8 and GBS-ds of 3 points as the cut-off for more severe impairment. Using the Fisher´s exact test a statistically significant association was found between high pre-treatment levels of IL8 and more severe impairment at nadir (p=0.0019) (**Figure 6**).

With the logistic regression model (**Table 7**) the same outcome as a cut-off for more severe impairment was used and levels of IL8 as both continuous and binary variables were investigated. Taking pre-treatment levels of IL8 as a binary variable, the same cut-off as above for high levels of IL8 (135 ng/L) was used. In the simple logistic regression model, IL8 as both a continuous and binary variable was associated with more severe impairment at nadir. In the multiple logistic regression model with age taken into consideration, a similar result was found, *i.e*., higher levels of IL8 were associated with more severe impairment at nadir.

**Table 7:** Logistic regression analysis of IL8 levels in cerebrospinal fluid (CSF) versus impairment at nadir in patients with GBS in the ELISA cohort.

Outcome	Variable	OR	95%CI	p	AUC	95%CI	p
Simple logistic regression							
nadir^1^	continuous IL8	1.04	1.01 – 1.08	0.03*	0.9	0.77 – 1.0	0.002*
	binary IL8^2^	40	4.3 – 1026	0.005*	0.86	0.69 – 1.0	0.006*
Multiple logistic regression							
nadir^1^	continuous IL8	1.04	1.01 – 1.09	0.02*	0.92	0.78 – 1.0	0.001*
	age	0.97	0.88 – 1.06	0.6	NA	NA	NA
naidr^1^	binary IL8^2^	44.6	4.03 – 2099	0.009*	0.86	0.69 – 1.0	0.005*
	age	0.99	0.89 – 1.09	0.8	NA	NA	NA

GBS, Guillain-Barré syndrome; GBS-ds, GBS disability scale; IL8, interleukin-8; OR, odds ratio; 95%CI, 95% confidence interval; AUC, area under curve; NA, not applicable.

^1^binary outcome mild versus severe impairment at nadir (severe impairment = GBS-ds 3 points or higher), ^2^ binary variable low versus high IL8 (cut-off based on GBS group median, 135 ng/L); *statistically significant.

Thus, the logistic regression analysis showed the same association between pre-treatment levels of IL8 and impairment at the nadir as Fisher´s exact test.

### IL8 and disease severity in patients with CIDP, PDN and MMN in the ELISA cohort

We found a positive correlation between pre-treatment levels of IL8 in patients with CIDP and disease severity measured with the INCAT scale only at the 18-month follow-up (Spearman r = 0.65, p = 0.008) (**Supplementary Figure 1** in the **Supplementary Material**). There was no correlation between IL8 levels and INCAT in patients with PDN and MMN.

### Correlation between IL8 and main laboratory findings in the ELISA cohort

There was no correlation between levels of IL8 and age, neither in all groups combined (r = 0.3, p < 0.0001), nor analyzed only in patients with inflammatory neuropathy (r = -0.01, p = 0.9) nor HC (r = 0.1, p = 0.5).

In patients with inflammatory neuropathies, we did not find any correlation between pre-treatment levels of IL8 and CSF cell counts (r = 0.01, p = 0.9), nor IgG-index (r = 0.3, p = 0.06). However, we found a positive correlation between pre-treatment levels of IL8 and albumin quotient (Q.alb) (CSF/plasma quotient of albumin) (r = 0.6, p < 0.0001), and with neurofilament light (NfL) (CSF r = 0.42, p = 0.01; plasma r = 0.5, p = 0.002). The correlation between IL8 and NfL was strongest in patients with CIDP (CSF r = 0.68, p = 0.003; plasma r = 0.62, p = 0.01). Of importance, five of the 21 patients with GBS with normal pre-treatment Q.alb had elevated levels of IL8 in CSF.

## Discussion

In this study, we investigated the diagnostic and prognostic value of IL8 in CSF and plasma in patients with acute and chronic inflammatory neuropathies. Moreover, our study reports, to our knowledge, the first data from high-throughput proteomics analyses for both acute and chronic inflammatory neuropathies using the PEA technology, resulting in discovery of potentially novel biomarkers.

Rigorous statistical analysis of multiplex data and validation with ELISA technique demonstrate that IL8 can be used in a diagnostic assessment of both GBS and CIDP in order to differentiate them from healthy individuals and from each other. Moreover, our results from the ELISA cohort indicate that IL8 could be used to differentiate patients with CIDP and PDN from those with non-inflammatory polyneuropathies (NIP), but due to the low number of patients with PDN, this should be further investigated in a larger cohort. Patients with MMN have predominantly distal nerve pathology, which could explain why we did not observe an increase in levels of IL8 in CSF in these patients. The data on levels of IL8 in patients with MMN and PDN, are to the best of our knowledge the first to be published.

In agreement with a recently published study (7), we confirm the IL8 concentration of 73 ng/L in CSF as a cut-off to differentiate patients with GBS from those with CIDP. However, due to the small sample size of patients with acute-onset CIDP (only 2 patients), we could not investigate if there was any statistical difference between patients with GBS and acute-onset CIDP. Of interest, all three patients with SIDP had values of IL8 higher than the cut-off and significantly higher compared to patients with CIDP. This observation indicates that IL8 may be used as a biomarker to differentiate acute as well as subacute inflammatory demyelinating neuropathies from chronic ones, but this needs to be verified in a larger cohort. Furthermore, this finding also suggests that levels of IL8 in CSF reflects the severity of inflammation of the nerve roots, which explains why the highest levels were found in patients with GBS. In addition, we suggest a novel finding of IL8 cut-off concentration at 51 ng/L in CSF to differentiate chronic demyelinating sensorimotor polyneuropathies (*i.e.*, CIDP and PDN) from non-inflammatory polyneuropathies.

Our study further suggests that levels of IL8 in CSF higher than 135 ng/L, when measured in the acute phase before treatment in patients with GBS, may predict a more severe impairment at nadir and a higher probability of progression in the acute phase of the disease. Based on these findings we propose that this group of GBS patients should be monitored more closely as they might further deteriorate. Furthermore, we report a possible prognostic value of IL8 in the long-term follow-up in GBS since pre-treatment levels of IL8 correlated with outcome as measured by GBS-ds at 6-months follow-up as well as with the EGOS score. Of note, in patients with CIDP we did not see a correlation between levels of IL8 and the outcome.

This study also highlights the use of PEA technology in search of novel biomarkers. The benefits of this technology are high sensitivity and specificity (15, 21), but such benefits may be offset by this method´s susceptibility to yield inconsistent results if sample handling and collection are not done in a strictly standardized way, yielding variability from protein degradation or protein leakage from blood cells (25, 26). In addition to IL8, and with the same rigorous statistical methods, we have identified six additional biomarkers that were upregulated in CSF in patients with GBS compared to HC (SELE, IL2RA, CR2, CD1C, THBD, and NRP1), and four compared with CIDP (ITGAM, IL2RA, IL6, and NRP1). The role of these biomarkers include the facilitation of cell-cell and/or cell-matrix adhesion (SELE, ITGAM, THBD; all expressed on monocytes) (27, 28), induction of the acute phase response (IL6) (29), T cell activation (IL2RA, CD1C) (30, 31), inhibition of B cell activation (CR2) (32), blood coagulation (THBD) (33), and a wide range of physiological and pathophysiological processes, such as immunity, cell migration, and angiogenesis (NRP1) (34).

In patients with CIDP, we also found an up-regulation of three proteins (MMP3, THBD, and ITGAM) in plasma compared to HC. MMP3 plays a role in tissue morphogenesis, cell migration, and angiogenesis, but it is also involved in the breakdown of the blood-nerve barrier and the pathological increase of its permeability (35, 36), which is a key event in pathogenesis of inflammatory neuropathies.

In conclusion, despite objective limitations of this study such as the retrospective design, the low number of patients and controls in the multiplex cohorts and a low number of patients with SIDP, acute-onset CIDP, PDN, and MMN in the ELISA cohort, we could confirm a previous report on IL8 in CSF as a diagnostic biomarker, specifically in patients with GBS and CIDP. Moreover, we could add new knowledge on IL8´s prognostic value, particularly in the acute phase of GBS. Our results suggest elevated levels of IL8 in CSF to be specific to inflammatory disorders of the peripheral nervous system such as GBS and CIDP, but not those of the central nervous system such as MS. In addition, and importantly, IL8 may provide an important marker for inflammatory neuropathies as opposed to non-inflammatory ones, which may have important implications in the work-up of neuropathies as well as for patient selection in treatment trials. The correlation between levels of IL8 and NfL in CSF further implicates intrathecal IL8 and/or IL8-secreeting cells such as monocytes in the pathogenesis of GBS and CIDP, though the exact role of IL8 remains unclear.

Using the PEA technology, we could also identify a number of potential biomarkers specific to GBS and CIDP, respectively. Some of these markers are predominantly secreted by monocytes, implicating the activation of the innate immune system in the pathogenesis of GBS and CIDP. Furthermore, upregulation of IL2RA in CSF in GBS, but not CIDP implicates activation of T-regulatory cells in the monophasic- (i.e., GBS), but not chronic inflammatory polyneuropathy, i.e., CIDP. Taken together, further longitudinal studies of the IL8 network are warranted as well as investigation and validation of the here proposed potentially novel diagnostic and prognostic biomarkers in patients with inflammatory neuropathies.

## Data availability statement

The original contributions presented in the study are included in the article/**Supplementary Material**, further inquiries can be directed to the corresponding author.

## Ethics statement

The studies involving humans were approved by Regional Ethical Review Board of Stockholm (EPN 2014/1815-31/4 and 2017/952–31/1) and Umeå Ethical Review Board (EPN 2011–39-31 M). The studies were conducted in accordance with the local legislation and institutional requirements. Due to the retrospective design of this study, the need for written informed consent was waived.

## Author contributions

Study conceptualization and design was done by IKm, RG, TO, MH, MA, and RP. IKm, KF, AS, KS, CI, and RP handled blood samples, recruited patients and controls and/or collected clinical data. IKm and IKo analyzed data. IKm wrote the manuscript and was responsible for making the figures and tables. RG, MA, and RP supervised the work. All authors contributed to the article and approved the submitted version.
