# Supplementary material for: Validation of elevated levels of interleukin-8 in the cerebrospinal fluid, and discovery of new biomarkers in patients with GBS and CIDP using a proximity extension assay
Source: Front Immunol. 2023 Nov 23;14:1241199. doi: 10.3389/fimmu.2023.1241199 (PMC10702497; doi:10.3389/fimmu.2023.1241199)
Supplement: Supplementary file 1 [file DataSheet_1.pdf]

Supplementary Material

Supplemental table S1. Call rate of all cerebrospinal fluid (CSF) and plasma proteins, their synonyms and main functions in multiplex cohorts.

PROTEIN	SYNONYM	MAIN FUNCTION	Olink discovery cohort call rate		Olink replication cohort call rate	
			CSF	plasma	CSF	plasma
**IL8**	Interleukin-8; CXCL8; 3-10C, AMCF-I (Alveolar Macrophage Chemotactic Factor-I), b-ENAP, GCP-1, GCP1, IL-8, IL8, K60, LECT, LUCT, LYNAP (Lymphocyte-Derived Neutrophil-Activating Peptide), MDNCF (Monocyte-Derived Neutrophil Chemotactic Factor), MONAP, NAF (Neutrophil Activation Factor), NAP-1, NAP1, SCYB8, TSG-1	Pro-inflammatory chemokine attracting neutrophils, other granulocytes, T cells, and dendritic cells to the site of inflammation. Produced mainly by blood monocytes, and tissue macrophages. Causes neutrophil activation and in synergy with vascular endothelial growth factor receptor 2 (VEGFR2) induces endothelial cell permeability.	100	100	100	100
**NCAM1**	Neural cell adhesion molecule 1; CD56 antigen; Leu19; NKH1	Cell-adhesion protein involved in neuron-neuron adhesion, axonal braching, neurogenesis, and expansion of T and B lymphocytes and natural killer cells.	100	100	100	100
**CD4**	CD4 antigen; T-cell surface glycoprotein CD4	Membrane glycoprotein of T lymphocytes. Acts as a coreceptor with the T-cell receptor on the T lymphocyte to recognize antigens displayed by an antigen presenting cell in the context of class II MHC molecules. In other cells such as macrophages or NK cells, plays a role in differentiation/activation, cytokine expression and cell migration.	100	100	45.5	100
**IL2RA**	Interleukin-2 receptor subunit alpha; CD25; IDDM10	Receptor for IL2. Involved in the regulation of immune tolerance by controlling regulatory T cells.	100	100	100	100
**CD5**	CD5 antigen; T-cell surface glycoprotein CD5; T1; Leu1	Glycoprotein expressed on all mature T-cells, thymocytes, and a subset of mature B-cells. May act as a receptor in regulating T-cell proliferation.	87.5	100	100	100
**CR2**	Complement C3d receptor type 2; CD 21 antigen; EBV receptor	Receptor for complement C3, for the Epstein-Barr virus on human B-cells and T-cells. Inhibition of B cell activation.	100	100	86.4	100

**CD38**	ADP ribosyl Cyclase 1; CD38 antigen; NIM-R5; ADPR cyclase T10; Lymphocyte Differentiation Antigen CD38	Transmembrane glycoprotein found on the surface of CD4+ and CD8+ T lymphocytes, B lymphocytes and natural killer cells. The CD38 protein is a marker of cell activation. It also functions in cell adhesion, signal transduction and calcium signaling.	100	100	100	100
**CD27**	CD27 antigen; T Cell Activation Antigen; S152; TNFRSF7 (Tumor Necrosis Factor Receptor Superfamily, Member 7); Tp55	Receptor for CD70/CD27L. May play a role in survival of activated T-cells and it´s required for generation and long-term maintenance of T cell immunity. It also plays a key role in regulating B-cell activation and immunoglobulin synthesis.	100	100	100	100
**CD40**	CD40 antigen; CD40L receptor; TNFRSF5 Receptor (Tumor necrosis factor receptor superfamily member 5); Bp50, p50	Receptor on antigen-presenting cells and it´s essential for mediating a broad variety of immune and inflammatory responses including T cell-dependent immunoglobulin class switching, and memory B cell development.	100	100	100	100
**KIT**	Mast/stem cell growth factor receptor Kit; C-Kit; CD117; PBT; SCFR	Cytokine receptor expressed on the surface of hematopoietic stem cells. It plays an essential role in the regulation of cell survival and proliferation, hematopoiesis, stem cell maintenance, gametogenesis, mast cell development, migration and function, and in melanogenesis. Signaling through KIT plays a role in cell survival, proliferation, and differentiation.	100	100	100	100
**IL3RA**	Interleukin-3 receptor subunit alpha; CD123 antigen	Receptor for IL3 expressed on hematopoietic progenitor cells, monocytes and B-lymphocytes that controls the production and differentiation of hematopoietic progenitor cells into lineage-restricted cells.	82.6	100	27.3	77.3
**CLEC4C**	C-type lectin domain family 4 member C; BDCA2; CD303; CLECSF11; CLECSF7; DLEC; HECL	Membrane protein of plasmacytoid dendritic cells; may play a role in antigen capturing by dendritic cells.	0	100	0	100
**NRP1**	Neuropilin-1; CD304; NRP protein; VEGF165R; Sema III	Cell-surface co-receptor for a number of extracellular ligands including				

	Receptor; Semaphorin III Receptor, A5 antigen; Npn 1 Protein	semaphorins, VEGFA and transforming growth factor beta. Has a role in a range of signaling pathways that utilize diverse extracellular ligands. Regulates VEGF-induced angiogenesis. Has a pleiotropic effect; affect cell survival, migration, and attraction.	100	100	86.4	100
**THBD**	Thrombomodulin; CD141; BDCA-3	Endothelial-specific membrane receptor that binds thrombin. This binding results in the activation of protein C, which degrades clotting factors Va and VIIIa and reduces the amount of thrombin generated and subsequently blood coagulation. It is also expressed on human mesothelial cell, monocyte and a dendritic cell subset.	100	100	100	100
**CD1C**	T-cell surface glycoprotein CD1c; CD1 antigen	Glycoproteins expressed on the surface of dendritic cells (DCs). CD1C proteins mediate the presentation of class I MHC antigen to T cells; they present lipids, glycolipids and small molecules antigens to T cells.	100	100	100	100
**ITGAM**	Integrin alpha-M; CD11b antigen; CR3A (Complement Component Receptor 3 alpha); MAC-1; Mo1 antigen; Mo1 glycoprotein	Protein subunit that forms with CD18 integrin alpha-M beta-2 (αMβ2) molecule, also known as macrophage-1 antigen (Mac-1) or complement receptor 3 (CR3). It is implicated in various adhesive interactions of monocytes, macrophages and granulocytes. It is also a receptor for fibrinogen, factor X and ICAM1, and regulates neutrophil migration.	8.3	50	90.9	100
**CCL3**	C-C motif chemokine 3; MIP 1 alpha (Macrophage Inflammatory Protein 1 alpha); G0S19-1; LD78 alpha chemokine; SCYA3; Small Inducible Cytokine A3; CCL3L3; CC Motif Ligand3-Like2; BB-10010	Pro-inflammatory cytokine involved in the acute inflammatory state in the recruitment and activation of polymorphonuclear leukocytes.	100	100	100	100
**CD28**	CD28 antigen; T-cell-specific surface glycoprotein CD28; TP44 receptor	Protein expressed on T cells that provide co-stimulatory signals required for T cell activation and survival, cytokine production, and T-helper type-2 development.	4.5	96.3	85.7	100

**PDCD1**	Programmed cell death protein 1; PD-1 protein; PD-1 receptor; CD279 antigen; hSLE1; SLEB2	Protein receptor expressed in activated T cells, B cells, NK and DC cells, macrophages and monocytes; it is involved in the regulation of T-cell functions and plays a critical role in induction and maintenance of immune tolerance to self-antigens (by down-regulating the immune system and promoting self-tolerance by suppressing T cell inflammatory activity).	4.2	96.2	22.7	100
**CD274**	Programmed cell death 1 ligand 1; PD-L1; PDL1; B7-H; B7-H1; B7H1 antigen; PDCD1LG1; CD274 antigen; B7H1 Immune Costimulatory Protein	Transmembrane glycoprotein expressed by macrophages, some activated T cells and B cells, DCs and some epithelial cells (and tumor cells). It binds to its receptor, PD-1 (=PDCD1), and modulates the activation of T-cells and limits T-cell effector response; i.e., together they play a role in preventing destructive autoimmunity, especially during inflammatory conditions (when PD-L1 binds PD-1 (a protein found on T cells), it keeps T cells from killing the PD-L1-containing cells, including the cancer cells).	100	100	100	100
**IL6**	Interleukin-6; BSF2 (B-Cell Stimulatory Factor 2), HGF (Hybridoma Growth Factor); HSF (Hepatocyte Stimulating Factor); IFNB2 (Interferon beta 2); B-Cell Differentiation Factor-2; Plasmacytoma Growth Factor; MGI-2	Pro-inflammatory cytokine (responsible for stimulating acute phase protein synthesis, as well as the production of neutrophils in the bone marrow, it supports the growth of B cells and is antagonistic to regulatory T cells) and an anti-inflammatory myokine (a cytokine produced from muscle, which is elevated in response to muscle contraction, it is thought to act in a hormone-like manner to mobilize extracellular substrates and/or augment substrate delivery).	100	100	100	100
**IL10**	Interleukin-10; CSIF (Cytokine Synthesis Inhibitory Factor); IL10A; TGIF	Anti-inflammatory cytokine, it targets antigen-presenting cells (APCs) such as macrophages and monocytes and inhibits their release of pro-inflammatory cytokines.	13.6	100	4.5	100
**IL12**	Interleukin-12 p70; T cell-stimulating factor	IL12A(p35) heterodimerizes with IL12B(p40) to form the IL12(p70) cytokine. It is involved in the differentiation of naive T cells into Th1 cells.	100	100	42.9	100

**IL17A**	Interleukin-17A; CTLA8	Pro-inflammatory cytokine produced by activated T cells (CD4+, CD8+, gamma-delta T (γδ-T), invariant NKT and innate lymphoid cells (ILCs) are primary sources of IL-17A). It promotes inflammation by inducing various proinflammatory cytokines and chemokines, recruiting neutrophils, enhancing antibody production, and activating T cells. IL-17A expression is also augmented in autoimmune diseases.	0	92.6	59.1	36.4
**IFNG**	Interferon gamma; Interferon Type II	Cytokin secreted by cells of both the innate and adaptive immune systems. It is an important activator of macrophage and it striggers a cellular response to viral and microbial infections.	0	11.1	13.6	81.8
**CEBPB**	CCAAT enhancer-binding protein beta; C/EBP-beta; CRP2; IL6DBP; LAP; NFIL6; TCF5	Transcription factor important for normal macrophage functioning.	NA	NA	40.9	40.9
**SELE**	E-selectin; Selectin E; CD62E antigen; ELAM; ELAM1 (Endothelial Leukocyte Adhesion Molecule-1); ESEL (Endothelial Leukocyte Adhesion Molecule 1); LECAM-2	Cell-surface glycoprotein found in cytokine-stimulated endothelial cells (= endothelial cells activated by cytokines) and is thought to be responsible for the accumulation of blood leukocytes at sites of inflammation by mediating the adhesion of cells to the vascular lining.	100	100	81.8	100
**MMP7**	Matrilysin; Matrix Metallopeptidase-7; MPSL1; PUMP-1;	Enzyme; it breaks down extracellular matrix (that provide structural and biochemical support to surrounding cells) by degrading macromolecules including casein, type I, II, IV, and V gelatins, fibronectin, and proteoglycan. The enzyme is involved in normal physiological processes, such as wound healing, as well as in disease processes, such as arthritis and metastasis.	100	100	50	100
**MMP9**	Matrix Metalloproteinase-9; CLG4B; 92-kDa Type IV Collagenase; Gelatinase B	Enzyme; it breaks down extracellular matrix (that provide structural and biochemical support to surrounding cells) by degrading type IV and type V collagen, and fibronectin. The enzyme is involved in	70.8	100	54.5	100

		normal physiological processes, such as wound healing, as well as in disease processes, such as arthritis and metastasis. Probably involved in IL-8-induced mobilization of hematopoietic progenitor cells from bone marrow.				
**MMP3**	Stromelysin-1; Matrix Metallopeptidase-3; STMY; STMY1; Transin	Enzyme; it breaks down extracellular matrix (that provide structural and biochemical support to surrounding cells) by degrading fibronectin, laminin, gelatins of type I, III, IV, and V; collagens III, IV, X, and IX, and cartilage proteoglycans. The enzyme is involved in normal physiological processes, such as wound healing, as well as in disease processes, such as arthritis and metastasis.	100	100	95.5	100
**CCL5**	C-C motif chemokine 5; D17S136E; MGC17164; T-cell RANTES protein; SCYA5; SISd; TCP228	Pro-inflammatory chemokine recruiting T cells, eosinophils, basophils, monocytes, natural-killer (NK) cells, dendritic cells and mastocytes to the site of inflammation. Produced mainly by T-cells and monocytes. Causes the release of histamine from basophils and activates eosinophils.	20.8	100	9.1	100

Thirteen Olink panels were analyzed:
Neurology, Neuroexploratory, Inflammation, Immune Response, Cardiometabolic, Cardiovascular II, Cardiovascular III, Cell Regulation, Development, Metabolism, Oncology II, Oncology III, and Organ Damage. Thirty-one markers were detected and passed QC for further analysis (proteins detected in less than 60% of the samples were excluded from further analyses).

Data on main function and synonyms is mainly based on The Human Protein Atlas (https://www.proteinatlas.org) and Swedish Medical Subject Heading (MeSH) database (https://mesh.kib.ki.se).

Abbreviations: CSF, cerebrospinal fluid

Supplemental figure S1. Correlation analysis in patients with chronic inflammatory demyelinating polyneuropathy (CIDP) in the ELISA cohort.

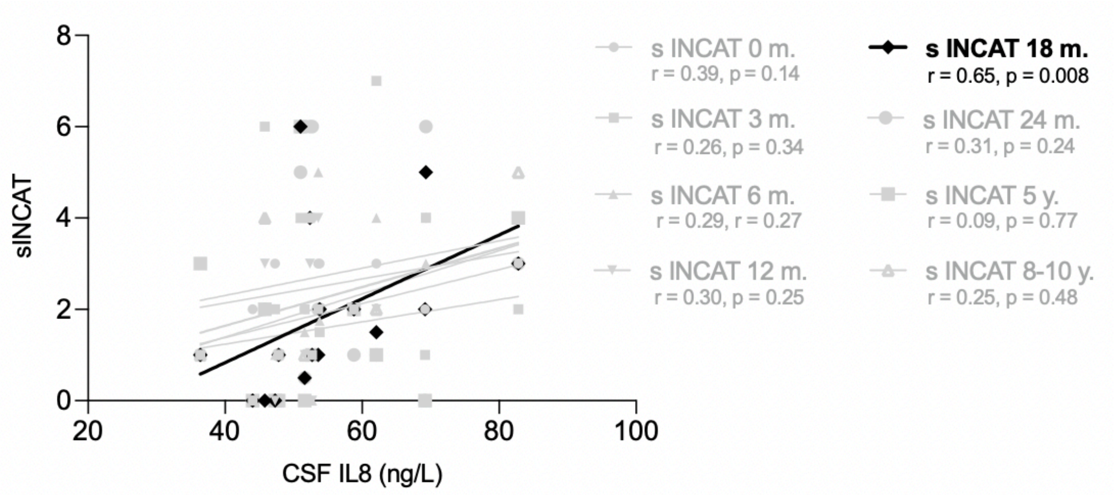

Abbreviations: CIDP, chronic inflammatory demyelinating polyneuropathy; sINCAT, sum score Inflammatory Neuropathy Cause and Treatment scale; r, Spearman correlation coefficient; CSF, cerebrospinal fluid; IL8, interleukin-8; m, months; y, years; 0 m, time of sampling; 3 m, 3-months follow-up; 6 m, 6-months follow-up etc.
